# COVID-19 vaccination and changes in preventive behaviours: findings from the 2021 vaccine roll-out in Switzerland

**DOI:** 10.1093/eurpub/ckad050

**Published:** 2023-04-04

**Authors:** Sara C Hitchman, Sarah Geber, Lukas Tribelhorn, Thomas N Friemel

**Affiliations:** Department of Communication and Media Research (IKMZ), University of Zurich, Zurich, Switzerland; Department of Communication and Media Research (IKMZ), University of Zurich, Zurich, Switzerland; Department of Communication and Media Research (IKMZ), University of Zurich, Zurich, Switzerland; Department of Communication and Media Research (IKMZ), University of Zurich, Zurich, Switzerland

## Abstract

**Background:**

Behavioural, environmental, social and systems interventions (BESSIs) remain important for controlling the COVID-19 pandemic in addition to vaccination. However, people’s adoption of BESSIs may decrease as vaccination rates increase due to reductions in the perceived threat of disease, and changes in risk perceptions of behaviours that increase the chance of infection. Thus, we examined predictors of and changes over time in reports of mask wearing and physical distancing and whether changes in mask wearing and physical distancing differed by vaccination status during the main 2021 COVID-19 vaccine roll-out period in Switzerland.

**Methods:**

Weekly online cross-sectional surveys (26 April 2021 to 1 August 2021) among people 18–79 years old in Switzerland, *N* = 6308 observations and 5511 cases. Logistic regression models using generalized estimating equations.

**Results:**

Reports of being vaccinated increased, while mask wearing and physical distancing decreased over time. This decrease was similar regardless of vaccination status. However, the level of reported mask wearing and physical distancing remained higher among vaccinated people. Older, female, and Italian language region respondents also had higher odds of reporting mask wearing and physical distancing.

**Conclusions:**

Adoption of COVID-19 preventive behaviours is associated with demographics and vaccination status. Further research is needed to understand the reasons why people who are not vaccinated are less likely to adopt preventive behaviours, including that they may have fewer social and environmental opportunities to do so.

## Introduction

Despite the introduction of a COVID-19 vaccine, non-pharmaceutical interventions (NPIs) or behavioural, environmental, social and systems interventions (BESSIs),^1^ such as masking and physical distancing remain important for controlling the COVID-19 pandemic.^2^ COVID-19 vaccination in Switzerland started in late December 2020 with the most vulnerable and began to rapidly progress in late April 2021 when vaccination started to be available to all adults.^3^ By the end of July 2021 the pace of vaccination had slowed.^3^ Although the vaccination rate continued to gradually increase after the end of July 2021, Switzerland currently has the lowest rate of COVID-19 vaccination in Western Europe, with only 67.5% of people vaccinated with two doses at the time of writing in January 2022,^4^ making the sustained adoption of BESSIs particularly critical in the Swiss context. Switzerland was administering the BNT162b2 and the mRNA-1273 vaccines at the time of this study.^5^

The challenge to sustaining BESSIs is that as an increasing number of people are vaccinated^6^ and governments lift preventive measures,^7^ the adoption of BESSIs may decrease to the point that a new wave of infections arrives.^2^ Theories of health behaviour suggest that the adoption of BESSIs may decrease under these circumstances. Most of these theories are organized around risk perceptions as central factor of preventive behaviour, such as the health belief model^8^ or the 5Cs vaccination model^9^ referring to complacency (i.e. an individual’s perception of risk of the disease) as a driver of vaccination uptake. Following this, it is reasonable to assume that getting vaccinated reduces risk perception and thus the motivation to engage in BESSIs. Comparably, the risk compensation hypothesis^10^^,^^11^ predicts that getting vaccinated against COVID-19 will lead people to assess behaviours that increase their chance of infection as less risky, leading to increased engagement in risky behaviours, potentially counteracting some of the benefits of vaccination.^11^ The risk compensation hypothesis has however been criticized in the context of COVID-19 preventive measures,^12^ and its application to other vaccinations,^13^ such as human papillomavirus vaccination (HPV). Finally, the Capability, Opportunity and Motivation model of behaviour (COM-B)^14^^,^^15^ suggests that people’s capabilities, opportunities and motivation to sustain COVID-19 preventive behaviours may be affected by a number of factors, such as their vaccination status and the prevention policies in place. For example, the motivation to wear a mask and physically distance may decrease once someone is vaccinated and believes they have reduced their own and others’ risk of severe infection,^16^ and opportunities to wear masks may decrease if mask wearing preventive measures are lifted.^16^

Despite concerns with changes in the adoption of preventive behaviours as vaccination rates increase and preventive measures are lifted, there is little research on the association between COVID-19 vaccination status and preventive behaviours over time. A study in UK examined online panel data with matched pairs of vaccinated and non-vaccinated people (October 2020 to March 2021) and found little evidence that people who had been vaccinated reduced compliance with preventive behaviours compared with people who were not vaccinated.^17^ For HPV, where there is more research, a systematic review of research studies overall found no evidence of riskier behaviours post-HPV vaccination, but that in some studies people who were vaccinated reported less risky behaviours compared with those who were not vaccinated.^13^

Thus, to add to our understanding of changes in adoption of COVID-19 preventive behaviours as vaccination rates increase and preventive measures are lifted, we examined changes in reports of consistent mask wearing and physical distancing during the main vaccine roll-out period in Switzerland using a weekly online survey during a 3-month period.

A previous UK study using similar methods (online weekly survey), found higher compliance with COVID-19 restrictions was associated with demographic factors including older age, female gender, geographic region, lower education/income and having no children in the home.^18^ A study in Switzerland conducted at earlier time points also found regional differences in adherence.^19^ Thus, we examined the association between these demographic characteristics and vaccination status, consistent mask wearing and physical distancing. We additionally included the presence of additional adults older than 65 years in the home to account for the people who live in homes with multiple older adults/multi-generational homes where the perceived risk of household members may be higher.^20^

### Masking and physical distancing preventive measures in Switzerland during the vaccination roll-out

People in Switzerland who were fully vaccinated (two doses at the time) were exempt from masking and physical distancing preventive measures when at private gatherings with other fully vaccinated people.^21^ However, they still needed to follow general preventive measures on masking and physical distancing.^21^

#### Masking

There was a general requirement to wear a mask in indoor places accessible to the public for people 12 years of age and older during the entire study period.^22^ Two significant preventive measures were lifted on the 26 June 2021 when masks at workplaces were no longer required in areas not publicly accessible, and when masks were no longer required in outdoor areas where distances could not be kept.^22^

#### Physical distancing

There was a general measure to keep at least 1.5 m apart from people outside your household throughout the study.^22^ Just prior to the study period, significant changes to rules that may have affected physical distancing occurred on 19 April 2021, when outdoor areas of restaurants re-opened, and events, sports, cultural activities and in-person teaching in tertiary education became possible again.^22^ The 31 May 2021, approximately 1 month into the study period, brought further significant changes, with the opening of indoor areas of restaurants, and an increase in capacity for events, sports and culture. The obligation to work from home was also lifted in cases where repetitive testing at work could be implemented, and replaced with a recommendation on 31 May 2021.^22^ Physical distancing restrictions were further eased 2 months into the study period on the 26 June 2021 as vaccination rates increased, and COVID-19 certificates began to be issued. Discos and dance venues re-opened but required a COVID-19 certificate documenting that people have had a COVID-19 vaccination, have recovered from an infection with COVID-19, or have a recent negative test result.^22^ People were able to return to workplaces without regular testing on 26 June 2021.

#### Research questions

To understand changes in preventive behaviours during the vaccine roll-out period in Switzerland we used weekly survey data and examined the following research questions: (i) What are the demographic characteristics of people who report that they are vaccinated? (ii) What are the demographic characteristics of people who report consistently wearing a mask and physical distancing? (iii) Were there changes in reports of consistently wearing a mask and physical distancing over time? (iv) Were there any differences in changes overtime in mask wearing and physical distancing between people who reported that they were vaccinated vs. not vaccinated.

## Methods

### Covid-Norms project

The ‘Covid-Norms’ project^23^ monitored COVID-19 protective behaviours, including mask wearing, physical distancing, and vaccination in Switzerland. The weekly online surveys are representative of people between 15- and 79-years old living in Switzerland, who use the internet at least once a week for personal purposes, and can complete a survey in German, French, or Italian. For this study, we used the weekly data from the period of 26 April to 1 August 2021 and from participants aged between 18 and 79 years old due to differences in vaccine availability across Cantons for people less than 18 years old.

### Sample

Samples for the weekly surveys were drawn from the LINK Internet Panel, part of YouGov.^24^ The LINK panel consists of 115 000 panelists in Switzerland. Panelists are recruited via registered landline numbers and randomly generated mobile numbers, thus the panel approaches a probability panel relative to the majority of online panels in Europe and North America who primarily use opt-in recruitment.^25^^,^^26^ Panelists were invited to participate in the survey by e-mail. Each weekly survey aimed for a sample of at least 425 respondents based on sampling quotas for language region, sex, and age. The same panel member could repeat multiple weekly surveys, however only if they have not participated in the previous 4 weeks. Younger and Italian region participants were more likely to repeat.

### Survey

Participants completed the online survey in German, French, or Italian, for the survey see link.^27^ Demographic information was sourced from the panel. Participants received points that they could exchange for vouchers, including supermarket gift cards, digital cash credit and charity donations. After completion of a checklist from the University of Zurich ethics committee, the study was exempted from full ethical review.

## Measures

### Preventive behaviours

#### Vaccination status

Participants were asked, ‘Have you been vaccinated against the coronavirus?’ with response options yes or no. No question was available to measure number of doses received.

#### Vaccination intentions

Participants who answered no to vaccination status were asked about their intentions to be vaccinated, ‘How likely are you to be vaccinated against the coronavirus?’ Participants gave responses on a 5-point Likert scale from 1 (*very unlikely*) to 5 (*very likely*). The variable was coded as intentions to be vaccinated,^4^^,^^5^ undecided,^3^ or no intentions to be vaccinated.^1^^,^^2^

#### Combined vaccination status and intentions

The vaccination status and intentions variables were then combined: vaccinated, intentions to be vaccinated, undecided and no intentions to be vaccinated.

#### Mask wearing

Participants were informed, ‘The next questions are about wearing a mask when it is not possible to keep a distance,’ and then asked: ‘How consistently do you wear a mask in these situations?’ Participants answered on a 5-point Likert Scale from 1 (*not at all consistently*) to 5 (*very consistently*). The variable was coded as not consistently^1–3^ vs. consistently.^4^^,^^5^

#### Physical distancing

Participants were informed, ‘The next questions are about keeping a distance from people who do not live in your household,’ and then asked: ‘How consistently do you try to keep a distance from people who do not live in your household?’ Participants answered on a 5-point Likert scale from 1 (not at all consistently) to 5 (very consistently). The variable was coded as not consistently^1–3^ vs. consistently.^4^^,^^5^

For the purposes of this study, we defined the adoption of mask wearing and physical distancing as reporting consistently engaging (scores of 4 and 5) in the behaviours because consistent adoption would be needed for the measures to work most effectively.

### Demographic characteristics

#### Age

Participants entered their age. Age was categorized as: 18–29, 30–44, 45–64, and 65 plus.

#### Sex

Participants entered their sex, male or female.

#### Education

Participants entered their highest level of education. This was grouped into low, medium, high, and no answer/refused.

#### Language region

Italian, French, or German.

#### Urbanity

Living in a primary urban city (e.g. Lausanne, Lugano, Zurich) vs. other.

#### Age of youngest child in household

Participants were asked the ages of any children in their home. Responses were coded as age of their youngest child: no children, 5 or under, 6–9, 10–14, or 15–19.

#### Adult household composition 65 and over

Participants were asked if other adults lived in their household and their age. Responses were coded as: single adult household, multiple adults under 65, multiple adults including adults 65 and over.

### Survey variables

#### Time/week

The 14 weeks of survey data were treated as a categorical variable ranging from 1 to 14, representing ISO calendar weeks 17–30 of 2021.

#### Number of surveys repeated (time-in-sample)

To account for some respondents repeating the weekly survey more than once, we adjusted for the number of survey waves that each respondent had completed since the beginning of the Covid-Norms project in September 2020 and up until the end of the current study period: 1, 2 or 3 or more surveys.

### Analyses

**Figure 1:**
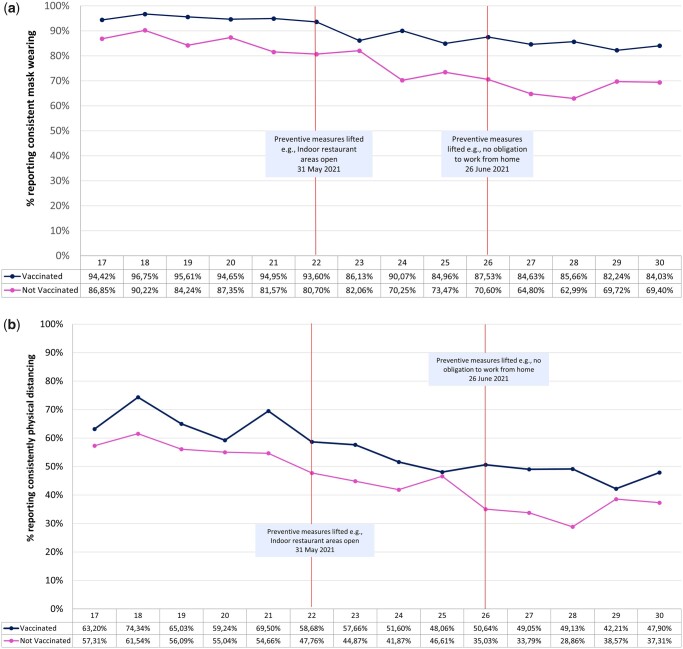
(a) Mask wearing by vaccination status during the main vaccine roll-out period in Switzerland (26 April 2021–1 August 2021). Group means of mask wearing over time by vaccination status controlled for demographic and survey variables. (b) Physical distancing by vaccination status during the main vaccine roll-out period in Switzerland (26 April 2021–1 August 2021). Group means of physical distancing over time by vaccination status controlled for demographic and survey variables.

Data were cleaned and coded in R version 4.1.0 and analyses were conducted in SAS 9.4, and were weighted (region, sex, and age) to be representative of people living in Switzerland. Logistic regression models using generalized estimating equations (GEEs) were used. Demographic characteristics and time-in-sample were treated as time in-variant, all other variables were time varying. In model 1, demographic predictors and survey variables were entered into a model to examine variables associated with being vaccinated vs. not vaccinated. In models 2 and 3, demographic variables, survey variables, and the combined vaccination status and intentions variable were entered into the models to test predictors of 2 consistent vs. not consistent mask wearing and 3 consistent vs. not consistent physical distancing. To test for differences in changes over time in mask wearing and physical distancing by vaccination status, the combined vaccination status and intention variable was replaced with the binary vaccination status in models 2 and 3 and the vaccination status*time interaction term was added. Group means of mask wearing and physical distancing over time by vaccination status were generated and presented in figure 1a and b controlling for variables in models 2 and 3.

## Results

### Sample

There were 6308 observations and 5511 cases in the final analytic sample. Of the 5511, 4789 completed the survey once, 647 twice, and 75 three times. See Supplemental figure S1 for study flow diagram and Supplemental table S1 for the number of participants in each weekly survey. Demographic characteristics of the 5511 cases are shown in table 1, most respondents were German-speaking, 45–64 years old, female, and had medium to high education. Further details of characteristics of the 6308 observations are in column 2 of Supplemental table S1.

**Table 1:** Demographic characteristics of sample (*N* = 5511 cases)

Characteristic	*n*	%
Language region		
Italian	1230	22.3%
German	2688	48.8%
French	1593	28.9%
Age		
18–29	1028	18.7%
30–44	1362	24.7%
45–64	2077	37.7%
65 and over	1044	18.9%
Sex		
Male	2697	48.9%
Female	2814	51.1%
Education		
Low	299	5.4%
Medium	2545	46.2%
High	2507	45.5%
Not reported	160	2.9%

### Preventive behaviours

#### Vaccination status

Older age, urban residence, high vs. low education, having completed one weekly survey vs. three surveys (less ‘time-in-sample’), and having an additional adult 65 and over in the home were associated with greater odds of being vaccinated vs. not. See Supplemental table S1. Using the last week of the study as the reference (week 30 ending 1 August), the vaccination rate increased at each week up until calendar week 24 (week ending 20 June), from week 25 onwards, there was no clear evidence that the vaccination rate was increasing in the sample. See Supplemental table S1 continued.

#### Consistent mask wearing

Being from the Italian vs. German/French language region, older age, being female vs. male, having no children vs. having a youngest child 15–19 years old, and being vaccinated vs. undecided or not intending to be vaccinated were associated with greater odds of reporting consistent mask wearing. See table 2. Reports of consistent mask wearing decreased overtime. Using the last week of the study as the reference (week 30 ending 1 August), consistently wearing a mask decreased at each week up until calendar week 22 (week ending 6 June), from week 23 onwards, there was no clear evidence that the odds of reporting consistent mask wearing was different than at week 30. See table 3. The vaccination status*time interaction was not significant (*P* = 0.522), thus there was no clear evidence that the declines found in mask wearing differed by vaccination status. See figure 1a for changes in mask wearing over time by vaccination status.

**Table 2:** Associations between respondent characteristics and consistent mask wearing during the main vaccine roll-out period in Switzerland, *N* = 6308 observations^a^

Characteristic	**% masks wearing** ^b^	OR	LCI	UCI	*P* value
Language region					
Italian	88.6	1.00	1.00	1.00	ref
German	82.4	0.58	0.46	0.73	<.0001
French	83.4	0.68	0.53	0.87	0.002
Age					
18–29	74.2	1.00	1.00	1.00	ref
30–44	79.0	1.30	1.00	1.68	0.049
45–64	88.1	2.17	1.69	2.80	<.0001
65 and over	93.4	4.12	2.77	6.13	<.0001
Sex					
Male	79.8	1.00	1.00	1.00	ref
Female	86.4	1.85	1.55	2.22	<.0001
Urban					
Urban	83.8	1.00	1.00	1.00	ref
Non-urban	82.7	0.99	0.81	1.23	0.960
Education					
Low	79.8	1.00	1.00	1.00	ref
Medium	82.6	1.10	0.75	1.61	0.637
High	83.6	1.04	0.71	1.54	0.839
Not reported	82.7	1.40	0.76	2.58	0.283
Number of waves completed					
1	86.4	1.00	1.00	1.00	ref
2	83.3	1.02	0.82	1.28	0.859
3 or more	75.3	0.87	0.68	1.11	0.273
Age of youngest child					
No children	82.8	1.00	1.00	1.00	ref
0–5 years	78.7	1.03	0.74	1.45	0.855
6–9 years	83.7	1.03	0.70	1.50	0.898
10–14 years	83.7	0.96	0.68	1.34	0.792
15–19 years	86.3	1.44	1.00	2.07	0.048
Adult household composition 65 and over					
Single adult household	82.9	1.00	1.00	1.00	ref
Multiple adults under 65	81.6	1.09	0.87	1.37	0.464
Multiple adults inc. 65 and over	88.3	0.93	0.66	1.31	0.691
Vaccination status					
Vaccinated	88.2	1.00	1.00	1.00	ref
No intention	58.8	0.18	0.15	0.22	<.0001
Undecided	75.6	0.44	0.33	0.59	<.0001
Intention	89.1	0.94	0.73	1.23	0.670

LCI: lower confidence interval; OR: odds ratio; UCI: upper confidence interval.

a Week/time was also adjusted for, see Table 3.

b Weighted percent pooled over weeks.

**Table 3:** Association between time/week and mask wearing during the main vaccine roll-out period in Switzerland, *N* = 6308^a^

Calendar week	**% mask wearing** ^b^	OR	LCI	UCI	*P* value
17—week starting 26 April 2021	87.3	2.25	1.44	3.52	<0.001
18	90.7	2.80	1.73	4.53	<.0001
19	85.6	1.87	1.20	2.91	0.005
20	89.3	2.16	1.39	3.37	<0.001
21	86.4	1.84	1.20	2.83	0.006
22—week starting 31 May 2021	86.2	1.82	1.18	2.78	0.006
23	83.0	1.30	0.83	2.03	0.246
24	80.5	1.19	0.80	1.76	0.396
25	79.4	1.04	0.70	1.53	0.856
26—week starting 28 June 2021	80.9	1.17	0.78	1.76	0.456
27	77.5	0.93	0.63	1.37	0.707
28	78.2	0.94	0.63	1.40	0.772
29	77.5	0.83	0.56	1.24	0.374
30—week ending 1 August 2021	78.2	1.00	1.00	1.00	ref

LCI: lower confidence interval; OR: odds ratio; UCI: upper confidence interval.

a Adjusted for covariates in Table 2.

b Weighted percentages.

#### Consistent physical distancing

Being from the Italian vs. German/French region, older age, female vs. male, having no children vs. children 10–14 years old, and being vaccinated vs. undecided or not intending to be vaccinated, were associated with greater odds of reporting consistent physical distancing. See table 4. Reports of physical distancing decreased over time. Using the last week of the study as the reference (week 30 ending 1 August), physical distancing decreased at each week up until calendar week 22 (week ending 6 June), from week 23 onwards, there was no clear evidence that the odds of reporting physical distancing at each week was different than at week 30. See table 5. The vaccination status*time interaction was not significant (*P* = 0.687), thus there was no clear evidence that the declines found in physical distancing differed by vaccination status. See figure 1b for changes in physical distancing over time by vaccination status.

**Table 4:** Associations between respondent characteristics and consistent physical distancing from people outside household during the main vaccine roll-out period in Switzerland, *N* = 6308 observations^a^

Characteristic	**% physical distancing** ^b^	OR	LCI	UCI	*P* value
Language region					
Italian	58.5	1.00	1.00	1.00	ref
German	51.8	0.80	0.68	0.93	0.005
French	50.2	0.76	0.64	0.90	0.001
Age					
18–29	29.8	1.00	1.00	1.00	ref
30–44	48.1	2.54	2.04	3.15	<.0001
45–64	62.1	4.33	3.54	5.29	<.0001
65 and over	73.9	7.12	5.44	9.33	<.0001
Sex					
Male	50.2	1.00	1.00	1.00	ref
Female	53.3	1.27	1.11	1.44	<0.001
Urban					
Urban	54.3	1.00	1.00	1.00	ref
Non-urban	50.9	0.94	0.81	1.10	0.466
Education					
Low	50.1	1.00	1.00	1.00	ref
Medium	51.1	0.83	0.62	1.11	0.206
High	52.7	0.78	0.58	1.04	0.095
Not reported	48.1	1.04	0.65	1.67	0.882
Number of waves completed					
1	57.7	1.00	1.00	1.00	ref
2	51.2	1.09	0.93	1.28	0.282
3 or more	39.7	1.15	0.95	1.40	0.147
Age of youngest child					
No children	52.3	1.00	1.00	1.00	ref
0–5 years	46.1	0.88	0.68	1.13	0.319
6–9 years	57.2	1.01	0.77	1.34	0.931
10–14 years	50.7	0.75	0.59	0.95	0.018
15–19 years	48.3	0.96	0.75	1.23	0.742
Adult household composition 65 and over					
Single adult household	52.8	1.00	1.00	1.00	ref
Multiple adults under 65	48.2	1.11	0.94	1.32	0.203
Multiple adults inc. 65 and over	64.1	1.04	0.82	1.31	0.763
Vaccination status					
Vaccinated	57.0	1.00	1.00	1.00	ref
No intention	31.1	0.38	0.31	0.46	<.0001
Undecided	39.3	0.61	0.48	0.78	<.0001
Intention	56.9	1.05	0.88	1.26	0.575

LCI: lower confidence interval; OR: odds ratio; UCI: upper confidence interval.

a Week/time was also adjusted for, see Table 5.

b Weighted percent pooled over weeks.

**Table 5:** Association between time/week and physical distancing during the main vaccine roll-out period in Switzerland, *N* = 6308^a^

Calendar week	**% physical distancing** ^b^	OR	LCI	UCI	*P* value
17—starting 26 April 2021	58.8	1.81	1.33	2.46	<0.001
18	63.7	2.19	1.59	3.00	<.0001
19	57.2	1.76	1.28	2.42	0.001
20	57.1	1.53	1.12	2.08	0.008
21	61.0	1.98	1.45	2.69	<.0001
22—week starting 31 May 2021	53.0	1.41	1.04	1.92	0.028
23	50.8	1.30	0.95	1.79	0.100
24	48.1	1.12	0.83	1.52	0.453
25	48.0	1.08	0.80	1.45	0.615
26—week starting 28 June 2021	45.9	1.04	0.77	1.41	0.780
27	45.7	0.99	0.73	1.34	0.943
28	44.8	0.94	0.70	1.28	0.713
29	43.5	0.82	0.60	1.11	0.201
30—week ending 1 August 2021	45.2	1.00	1.00	1.00	ref

LCI: lower confidence interval; OR: odds ratio; UCI: upper confidence interval.

a Adjusted for covariates in Table 4.

b Weighted percentages.

## Discussion

This is the first in-depth study of how preventive behaviours changed in the Swiss population during the main COVID-19 vaccine roll-out period. We found getting vaccinated was associated with older age, living in a primary urban area, having higher vs. lower education, and living with other adults 65 years old and over. For preventive behaviours, we found that key demographic characteristics were associated with higher odds of being more likely to adopt mask wearing and physical distancing behaviours, including being from the Italian vs. German/French language regions, being older and female. People who were vaccinated and intending to be vaccinated also had higher odds of consistent mask wearing and physical distancing behaviours throughout the study period. We also found that in comparison to the end of the current study period (1 August 2021), that odds of reporting mask wearing and physical distancing were higher until calendar week 22 (week ending 6 June 2021), which coincided with the date when some preventive measures were lifted by the Swiss government on 31 May 2021 (e.g. indoor areas of restaurants re-opened and obligation to work from home lifted with testing). We did not intend to study the specific effects of the lifting of preventive measures so this finding should be considered exploratory. There was no clear evidence of differences in changes over time in either mask wearing or physical distancing by vaccination status, both groups had higher odds of mask wearing and physical distancing at the beginning compared with the end of the vaccine roll-out period, and people who were not vaccinated were consistently less likely to adopt preventive behaviours.

For mask wearing and physical distancing, our findings are similar to previous studies that have found characteristics including being male and younger to be associated with lower self-reported compliance with COVID-19 measures.^18^^,^^28^ The finding that mask wearing and physical distancing were higher in Italian vs. German/French language region respondents is similar to previous studies.^19^ Regional differences may be due to discussions in media,^29^ political leaning,^30^ and perceived severity of COVID-19.^31^ Perceived severity of COVID-19 may be higher in the Italian region (Ticino) because neighbouring Lombardy is the first area where the pandemic emerged and a large number of deaths occurred in Europe.^29^ The finding that preventive behaviours did not decrease at a greater rate among the vaccinated compared with the not vaccinated is similar to previous studies that have not found greater risk taking behaviours after vaccination^13^^,^^17^ and provides further evidence against the risk compensation theory.^32^

Generally, our findings indicate that perceptions of threat or complacency (as defined in the 5Cs vaccination model^9^) are stable perceptions that are linked to the disease, independently of people’s vaccination status. This is fortunate in that vulnerable groups (e.g. elderly) appear to use all available preventive behaviours to reduce their risk; this is unfortunate in that there are groups of people who tend to feel generally less threatened and engage less consistently in preventive behaviours regardless of their vaccination status.

### Limitations

First, the measure of vaccination status was a yes or no measure, thus we could not ascertain whether someone was fully protected. This would have required asking whether they had received one dose or two doses and the time since the second dose. Second, we relied on self-reported measures of behaviour, thus it is possible that people may have been more likely to give responses in line with what they perceived to be socially desirable. Despite this, the characteristics we found to be associated with adopting preventive behaviours were similar to previous studies, and the adoption of preventive behaviours decreased overtime in line with the lifting of preventive measures and increasing vaccination rates as expected. Furthermore, a potential bias by self-reporting would only affect our results if it would interact with the variables under investigation. Third, our sample was drawn from an online panel sample, and thus only represents people who use the internet and can complete a survey in German, French, or Italian. People without a phone line or mobile phone, and who are institutionalized are not represented. The vaccination rate in our sample was also higher than the rate reported in official vaccination statistics, suggesting that it likely underrepresents groups such as younger people with lower vaccination rates. It is also important to acknowledge that having the social and environmental opportunities to wear masks and physically distance is important,^14^ thus unmeasured confounders, such as employment in an industry that does not allow working from home, or a work place that does not support/enforce mask wearing or physically distancing measures could have affected our results. Lastly, no question was asked about previous infections. A previous infection could influence risk perceptions of a subsequent infection, vaccination decisions, or masking/physical distancing.

## Conclusion

Consistent mask wearing and physical distancing is lower among people who are not vaccinated. This is concerning as people who are not vaccinated are more likely to catch and transmit SARS-CoV-2, and in the absence of vaccination it is even more important that they adopt preventive behaviours to protect themselves and others. Further research is needed to understand where people who are not vaccinated may have gaps in their capabilities and opportunities to wear masks and practice physical distancing.^14^

## Supplementary Material

ckad050_Supplementary_Data

## Data Availability

Data and code are available at reasonable request. Reporting consistent mask wearing and physical distancing decreased overtime during the main vaccine roll-out period in Switzerland, which coincided with an increase in the percentage of the population that was vaccinated and the lifting of preventive measures. Reporting consistent mask wearing and physical distancing was associated with older age, being female, being vaccinated, and being from the Italian language region of Switzerland vs. the German and French regions. There was no clear evidence of a difference in changes over time in mask wearing and physical distancing by vaccination status, adding to the evidence base against the risk compensation theory; vaccinated people had higher reports of consistent mask wearing and physical distancing throughout the study period. Lower levels of consistent mask wearing and physical distancing among people who are not vaccinated highlight the urgent need for research on gaps in people’s capabilities and opportunities to wear masks and practice physical distancing, particularly among people who are not vaccinated.
