# Supplementary material for: COVID-19 vaccination and changes in preventive behaviours: findings from the 2021 vaccine roll-out in Switzerland
Source: Eur J Public Health. 2023 Apr 4;33(3):482–9. doi: 10.1093/eurpub/ckad050 (PMC10234639; doi:10.1093/eurpub/ckad050)
Supplement: ckad050_Supplementary_Data [file ckad050_supplementary_data.docx]

***Supplemental Figure 1.* Study Flow Diagram**

Removed 10 observations due to inconsistent age across waves

Selected for ISO calendar weeks 17-30 of 2021

Removed N=3 observations with missing household composition information

Removed 4 observations with residence in Lichtenstein and 1 with missing Canton

Removed N=31 observations less than 18 years

Sample at ISO week 35, N=22,982 observations

N=22972 observations

N=6347 observations

N=6342 observations

N=6311 observations

Final analytic sample, N=6308 observations

| ***Supplemental Table 1.* Associations between repsondent characteristics and vaccination status during the main vaccine roll-out period in Switzerland, N=6308 observations** | | | | | | |
| --- | --- | --- | --- | --- | --- | --- |
| **Characteristic** | **N†** | **% vaccinated‡** | **OR** | **LCI** | **UCI** | **p-value** |
| Language region |  |  |  |  |  |  |
| Italian | 1519 | 53,38 | 1,00 | 1,00 | 1,00 | ref |
| German | 2935 | 57,61 | 1,07 | 0,92 | 1,26 | 0,383 |
| French | 1854 | 54,74 | 1,02 | 0,86 | 1,21 | 0,824 |
| Age |  |  |  |  |  |  |
| 18-29 | 1449 | 42,97 | 1,00 | 1,00 | 1,00 | ref |
| 30-44 | 1550 | 46,47 | 1,05 | 0,84 | 1,31 | 0,646 |
| 45-64 | 2234 | 60,98 | 2,02 | 1,65 | 2,48 | <.0001 |
| 65 and over | 1075 | 86,59 | 8,57 | 6,19 | 11,85 | <.0001 |
| Sex |  |  |  |  |  |  |
| Male | 3207 | 58,30 | 1,00 | 1,00 | 1,00 | ref |
| Female | 3101 | 54,85 | 0,91 | 0,79 | 1,05 | 0,183 |
| Urban |  |  |  |  |  |  |
| Urban | 1560 | 60,04 | 1,00 | 1,00 | 1,00 | ref |
| Non-urban | 4748 | 55,69 | 0,83 | 0,70 | 0,98 | 0,024 |
| Education |  |  |  |  |  |  |
| Low | 342 | 46,30 | 1,00 | 1,00 | 1,00 | ref |
| Medium | 2922 | 53,48 | 1,31 | 0,95 | 1,82 | 0,104 |
| High | 2863 | 61,36 | 2,05 | 1,47 | 2,85 | <.0001 |
| Not reported | 181 | 55,51 | 1,39 | 0,84 | 2,33 | 0,2041 |
| Number of waves completed |  |  |  |  |  |  |
| 1 | 2966 | 63,47 | 1,00 | 1,00 | 1,00 | ref |
| 2 | 1553 | 54,33 | 0,94 | 0,79 | 1,12 | 0,466 |
| 3 or more | 1789 | 44,89 | 0,78 | 0,64 | 0,95 | 0,015 |
| Age of youngest child |  |  |  |  |  |  |
| No children | 4443 | 59,54 | 1,00 | 1,00 | 1,00 | ref |
| 0-5 | 473 | 47,27 | 0,77 | 0,59 | 1,03 | 0,074 |
| 6-9 years | 376 | 48,47 | 0,78 | 0,58 | 1,05 | 0,098 |
| 10-14 years | 495 | 51,53 | 0,81 | 0,63 | 1,05 | 0,115 |
| 15-19 years | 521 | 50,15 | 0,90 | 0,70 | 1,15 | 0,385 |
| Adult household composition 65 and over | |  |  |  |  |  |
| Single adult household | 1367 | 56,74 | 1,00 | 1,00 | 1,00 | ref |
| Multiple adults under 65 | 3914 | 51,44 | 1,07 | 0,90 | 1,28 | 0,439 |
| Multiple adults inc. 65 and over | 1027 | 78,17 | 1,55 | 1,18 | 2,03 | 0,002 |
| *Week/time was also adjusted for, see Supplemental Table 1 continued. | | | | | | |
| †unweighted sample size of total observations | | |  |  |  |  |
| ‡weighted percent pooled over weeks | |  |  |  |  |  |

| ***Supplmental Table 1 continued.* Association between time/week and vaccination status during the main vaccine roll-out period in Switzerland, N=6308*** | | | | | | |
| --- | --- | --- | --- | --- | --- | --- |
| **Calendar Week** | **N†** | **% vaccinated‡** | **OR** | **LCI** | **UCI** | **p-value** |
| 17 - week starting 26 April 2021 | 456 | 27,06 | 0,09 | 0,06 | 0,12 | <.0001 |
| 18 | 455 | 30,89 | 0,11 | 0,08 | 0,15 | <.0001 |
| 19 | 451 | 32,50 | 0,12 | 0,08 | 0,17 | <.0001 |
| 20 | 451 | 45,74 | 0,22 | 0,16 | 0,30 | <.0001 |
| 21 | 451 | 44,84 | 0,22 | 0,16 | 0,31 | <.0001 |
| 22 - week starting 31 May 2021 | 443 | 53,74 | 0,32 | 0,23 | 0,45 | <.0001 |
| 23 | 451 | 58,21 | 0,43 | 0,31 | 0,60 | <.0001 |
| 24 | 452 | 62,41 | 0,52 | 0,37 | 0,72 | <.0001 |
| 25 | 451 | 70,61 | 0,79 | 0,57 | 1,11 | 0,180 |
| 26 - week starting 28 June 2021 | 447 | 72,28 | 0,89 | 0,63 | 1,27 | 0,528 |
| 27 | 449 | 72,50 | 0,90 | 0,63 | 1,29 | 0,568 |
| 28 | 455 | 75,24 | 1,00 | 0,70 | 1,42 | 0,994 |
| 29 | 444 | 73,76 | 0,90 | 0,63 | 1,29 | 0,569 |
| 30 - week ending 1 August 2021 | 452 | 74,51 | 1,00 | 1,00 | 1,00 | ref |
| *Continuation of Table 1, adjusted for covariates in supplmental Table 1. | | | |  |  |  |
| †unweighted frequency |  |  |  |  |  |  |
| ‡weighted percent |  |  |  |  |  |  |
